# ‘It is never-ending work’: Colombian occupational therapists’ experiences of working in stroke rehabilitation

**DOI:** 10.1177/03080226231178396

**Published:** 2023-06-12

**Authors:** Karen Aguia-Rojas, Robert Martyn Bracewell, Juan Alberto Castillo, Jonathon O’Brien

**Affiliations:** 1 School of Medicine and Health Sciences, Universidad del Rosario, Bogotá, D.C., Colombia; 2 Bangor University, Bangor, UK; 3 School of Health Sciences, University of Liverpool, Liverpool, UK

**Keywords:** Stroke, Colombia, services, health care quality

## Abstract

**Introduction:**

Stroke is a leading cause of death and impairment in Colombia. There is unequal access to occupational therapy for stroke survivors and few data on services provided by occupational therapists. The study explored the experiences of Colombian occupational therapists working in stroke rehabilitation.

**Method:**

A total of 11 occupational therapists involved in the stroke rehabilitation in Colombia took part in two focus groups. Responses were analysed using thematic analysis within a critical realist theoretical framework.

**Findings:**

Three main themes were identified. First, participants discussed the ‘occupational therapy service’, highlighting changes since the 1970s, examples of contemporary good practice, and a range of barriers. Second, ‘attitudes’ included the impact of other professions views of occupational therapy and also the effects of low pay. Third, ‘action/way forward’ involved proposals for improving the current situation.

**Conclusion:**

Occupational therapists engaged in stroke rehabilitation in Colombia report a number of barriers. However, some draw inspiration from their past while others stress some strengths in current services. Future research should focus on occupational therapy stroke rehabilitation in Colombia’s regions and explore the impact of conflict and post-conflict contexts. In addition, occupational therapists’ efforts to improve professional representation should be explored.

## Background

In Colombia, cerebrovascular diseases are responsible for 23.5% of deaths, and stroke is the third cause of death (Arenas Duque and Lucumí, 2019). In addition, the economic burden of stroke in Colombia is high, and Camacho et al. (2020) have estimated that the presence of stroke increases a person’s care costs by 10–16 times. Colombian stroke survivors have significantly poorer scores on a range of quality of life measures, when compared with a non-stroke sample, having implications for occupational performance, including instrumental activities of daily living and social participation (Calderón-Chagualá et al., 2015).

The current Colombian health system was created by Law 100 in 1993 and requires all citizens to affiliate to the General System of Social Security in Health (SGSSS) via ‘Entidades Promotoras de Salud’ (EPSs), which are companies that offer varied healthcare plans through health providers called ‘Instituciones Prestadoras de Servicios’ (IPS) (Guerrero et al., 2011). Furthermore, the National Development Plan 2018–2022 states that there must be a guarantee of universal health coverage. Therefore, when a person is not affiliated to the SGSSS, they are affiliated with a health regimen according to their payment capacity. The population that is unable to pay will be affiliated with the subsidiary regimen and receive a complete subsidy (Colombian Government Ley 1955, 2019). However, individuals belonging to the subsidised health insurance scheme have poorer access to stroke services and higher rates of post-stroke mortality, and it has also been shown that most of the stroke provision is directed towards those in the contributory schemes (Arenas Duque and Lucumí, 2019; Camacho et al., 2018). Overall, in Colombia, stroke survivors living in vulnerable conditions have more severe disabilities and lower access to treatment and health services (Henao-Lema and Arcos-Rodríguez, 2020).

Another factor specific to Colombia is the recent history of armed conflict in the country, leading to the most serious humanitarian crisis in Latin America, and the second highest level of internally displaced people in the world (Peñas Felizzola et al., 2015). This background may also impact stroke. For example, in one area, Valle de Cauca, a region markedly affected by internal displacement due to paramilitary violence (Marín, 2006), the incidence of stroke increased by 58% between 2010 and 2011 (Agredo et al., 2013). Armed conflict has been linked to higher stroke incidence, with possible mechanisms being increased blood pressure and greater tobacco and alcohol use (Jawad et al., 2019). These last two behavioural risk factors have been specifically reported in Colombian conflict regions (Gómez-Restrepo et al., 2016).

## Literature review

The effectiveness of occupational therapy in maintaining the independence of stroke survivors, and even in reducing post-stroke mortality, has been demonstrated by Legg et al. (2007). In addition, the Royal College of Physicians (2016) in the United Kingdom (UK) recommend that occupational therapists, as part of the multidisciplinary team, should assess and treat problems in occupational performance areas, including activities of daily living, cognition and transition from hospital. Despite this, Colombian stroke survivors have limited access to occupational therapy and there is a general gap in health services, with some lower resourced rural regions experiencing restricted access, alongside greater stroke mortality levels in urban areas and an overall need for more stroke data (Calderón-Chagualá et al., 2015; Guerrero Agámez et al., 2021; Yanez et al., 2020). Indeed, there are currently no data in Colombia about stroke rehabilitation available following discharge from the hospital or about the intensity of rehabilitation in the community, and there is even doubt about the precise number of hospital-based multidisciplinary stroke units in the country (Bayona-Ortiz et al., 2019; Ouriques Martins et al., 2019). We identified a lack of information on the experiences of occupational therapists specialising in stroke rehabilitation in Colombia, and therefore investigated these using a qualitative methodology, as part of the project ‘Stroke rehabilitation in Colombia: helping to develop networks and capacity’. The aim of the project was to identify priorities for occupational therapy in stroke rehabilitation in Colombia and strengthen the links between universities in Colombia and the UK. It was supported by Official Development Assistance, a United Kingdom Foreign, Commonwealth and Development Office fund. Our research question was ‘what are the experiences of occupational therapists working in stroke rehabilitation in Colombia?’.

## Method

### Sampling

We used a sample of 11 individuals, divided into two focus groups. This is in line with evidence that optimal numbers for focus groups, in relation to the generation of ideas by discussants, range from 4 to 12 (Asquith, 1997).

A purposive sampling approach (Charmaz, 2011) was used, with participants being recruited from contacts of the occupational therapy department at Universidad del Rosario, Bogotá, D.C. Our inclusion criterion was that all participants should have clinical working experience, as part of a current or previous paid role, in stroke rehabilitation in Colombia. There are very few Colombian occupational therapists specialising in stroke rehabilitation and clinical roles tend to be more generic, so it was not possible to specify a time criterion for this clinical experience. However, our sample met the requirements of a purposively selected sample, in that the participants were all able to contribute information relevant to our research question (Etikan et al., 2016). All participants were of Colombian nationality and spoke Spanish as a first language.

### Data collection

Two focus group discussions were conducted. The first group consisted of five occupational therapists, all of whom had experience of both clinical practice in stroke and university teaching on occupational therapy programmes. The second group consisted of six occupational therapists, all of whom were in solely clinical roles. The details of participants are given in Tables 1 and 2.

**Table 1.** Focus group 1 details.

Name	Status
Carmen	Retired clinician
	Previously lectured in occupational therapy for 32 years
	Currently works as a university clinical instructor
Daniela	Forty years of experience in occupational therapy university teaching and clinical practice
	Specialist in treatment of older adults
Candelaria	Seven years of clinical experience with specialism in neurosciences
	Clinical teaching role in university hospital
Consuelo	Two years of experience as lecturer in occupational therapy
	Specialist in neurodevelopment
	Maintains clinical practice with children and adults
Rosa	Hospital-based clinician and university lecturer in occupational therapy
	Specialist in work-based rehabilitation and university teaching

**Table 2.** Focus group 2 details.

Name	Status
Paola	Clinician in military hospital
Alejandra	Clinician in hospital-based critical care centre for stroke
Valentina	Clinician in university clinic
Laura	Hospital-based clinician with 25 years of experience
Lina	Hospital-based clinician with 15 years of experience
Camila	Hospital-based clinician with 15 years of experience

The questionnaire for each group included preliminary, key, transition and concluding questions. The same schedule was used for both groups. The questions were asked by JO’B, while another member of the Universidad del Rosario occupational therapy department was also present as an observer in each group.

### Data analysis

The focus group responses were recorded and transcribed. Thematic analysis, as outlined by Braun and Clarke (2006) was used to identify themes. First, the Spanish language transcripts were read repeatedly to identify primary codes. From these codes, themes were identified. These were then grouped into broader themes and visual ‘thematic maps’ were constructed that illustrated the overarching themes (Braun and Clarke, 2006: 89). These themes were named and a series of example illustrative quotes were selected. The quotes were then translated into English by JO’B. The thematic analysis was an iterative process and involved constant referral between two of the researchers (JO’B and KAR), who reached an agreement on the themes before moving to the next stage.

When using thematic analysis, we were mindful of the recommendation to develop ‘meaning-based interpretative stories’ (Braun and Clark, 2023: 3), as opposed to merely generating named themes that could, feasibly, have been predicted prior to data analysis. For this reason, we did not have a predetermined hypothesis.

### Translation

The recorded focus groups were initially transcribed in Spanish by a professional translator who was a native Spanish speaker. We then used the technique of ‘back-translation’, which is recommended by Marshall (2007: 31) as a way to support validity. One researcher (JO’B) translated the participants’ quotes chosen to support thematic analysis into English from the original Spanish. Another researcher (KAR) then ‘back-translated’ these translations into Spanish. We then compared the back-translations with the original quotes in Spanish to check for accuracy. Any variation was then discussed between the researchers and a consensus was reached on the final English translation.

### Theoretical framework

We took a critical realist approach to our data analysis. We felt that critical realism’s view of the person as an open ‘laminated system’, shaped by multiple factors, including the psychosocial, socioeconomic and cultural, fitted well with our aim of exploring occupational therapists’ experiences within a specific service delivery context (Bhaskar and Hartwig, 2016: 278). We also felt that thematic analysis complemented the critical realist framework, in that thematic analysis fits both realist and constructionist theoretical approaches, which are combined in critical realism (Bhaskar and Hartwig, 2016; Braun and Clarke, 2006).

### Ethics

Ethical approval was granted by the University of Liverpool Health and Life Sciences Research Ethics Committee and the Universidad del Rosario Life Sciences Research Ethics Committee. All participants were asked to read and sign an informed consent document prior to taking part in the research.

## Findings

We identified three overarching themes: ‘the occupational therapy service’; ‘attitudes’ and ‘action/way forward’.

### The occupational therapy service

Six respondents (Alejandra, Daniela, Valentina, Laura, Lina, Camila) described positive experience of working in occupational therapy stroke services in Colombia. Daniela recalled working in stroke rehabilitation from the 1970s up until 2000:

I worked with nursing. . .it was nice work because the occupational therapy intervention was started at once. . . from my hospital service we went to the houses on home visits. . . the treatment was complete, integral, because it was not just occupational therapy. . .it makes human work, individual, specific for each one. That is what we have lost and what I think we have to rescue.

Valentina echoed this in her description of her current clinical role:

We have a process that is like the strength of the. . .clinic, which is the integral rehabilitation process.

Other occupational therapists (Lina and Alejandra) were currently working in what they described as ‘a centre of excellence’ for stroke care in Bogotá. This was characterised by occupational therapy assessment within 24 hours of admission, a multidisciplinary approach, and consistent occupational therapy input throughout the person’s time in the centre. Alejandra, for example, stated:

We do the rehabilitation early. . .we do not lose the patient. . . we are there all the time, one hundred per cent with the patient.

Moreover, Alejandra stressed the autonomy and equality of occupational therapists within the stroke team:

We are the ones who decide. . .like each of the team members.

These comments were not, however, representative of the whole discussion, and other participants perceived failings in their services. Daniela recalled that:

They closed the hospital. . .and the (patients’) stay was very short. . .they practically left in an acute state, it was very sad and painful.

Other participants (Daniela, Rosa, Consuelo, Candelaria, Lina) made similar complaints about premature discharge of stroke survivors along with delays in arrival to community-based occupational therapy services. Consuelo, for example, commented:

I think that one of the main challenges is precisely that they are leaving in this acute state from hospital and they arrive at the external service with one- or two-months delay, which is very valuable time lost for rehabilitation.

Candelaria worked in an outpatient service and described seeing patients

Who left the hospital two months or two and half months (previously).

Laura commented that this had created a general problem:

I think the part (where) we have lost a lot of terrain is in the outpatient part.

The EPS health insurers were felt to be responsible both for premature transfer of care and for the delays in seeing patients who had been discharged from hospital, and were criticised by several participants (Candelaria, Consuelo, Rosa, Lina). Lina commented:

There are delays in the outpatient rehabilitation, while the therapy service is authorized.

While Candelaria explained that, in relation to stroke survivors:

The EPS packages are very rigid and they say ‘no, give them one or two times a week’ and practically we lose all the work that has been done in the hospital and the patients start to regress.

The role of the EPS also meant that, in some cases, stroke survivors were not seen while in hospital. Alejandra stated:

We have 72% of compliance with occupational therapy, the rest don’t have adherence to treatment because of difficulties with the health insurers.

While Camila commented that:

If we have (patients) they are also followed up as outpatients, but it was the same, it depends on the insurer.

Another participant, Valentina, explained that:

Generally, the health insurers include check-ups by medics and physiotherapists, but not occupational therapy.

### Attitudes

Several participants (Candelaria, Paola, Lina, Camila) talked about the lack of understanding of the role of occupational therapy within the multidisciplinary stroke team. Camila stated:

We talk about neurologists, of physiatrists. . .and unfortunately (there is) lack of knowledge of the work we do. . .as occupational therapy.

An interesting sub-theme was the association of occupational therapists with childlike activity. Paola, for example, talked of the need to:

Change this perspective they have (that the) therapist comes to play, entertain, the nursing assistant comes in ‘you are playing? I’ll come later’.

She described a clinical situation where another professional asked:

‘Ah, you are playing?’ but they did not understand that we were working on managing visual-spatial skills, or that we were working at the beginning of hemianopsia, they didn’t understand any of these things we were working on.

Valentina stated that other health personnel believe:

The therapist is the one who works with children and that’s it. . .if there is difficulty, a neurological alteration, no, the physio works on this.

Explanations were offered for this lack of knowledge of occupational therapy in stroke teams by Camila:

The majority of medics don’t know what it is unless they have experienced it.

This participant also highlighted that:

The majority of our bosses are physiotherapists, so they don’t know what we do and they don’t involve us in the group.

Lina, working in the ‘centre of excellence’, described how the occupational therapists in her service had successfully met some of these challenges:

Before (the centre was started) they prepared meetings that took place every Thursday in which, apart from being a means of communication where the patient with a stroke code arrived for everyone, everyone was very clear about what the occupational therapist does, all the residents on rotation who are passing through, all are clear that first they need to refer to physiotherapy, social work, and also to occupational therapy.

Another issue was that occupational therapists were not attracted to clinical work. One participant in group two (Camila) commented:

All the time there are less occupational therapists who work clinically, I don’t know why, there is lack of motivation, lack of interest concerning the clinic and it is very sad.

Camila felt that one issue here was poor remuneration, which made it harder to attract students to clinical work:

Here, unfortunately, I believe it is not possible to get occupational therapists for every hospital because of the pay they receive. Unfortunately, they go to other sectors. . .to make therapists fall in love so that they come here is difficult. I say to all the students from the same University in the hospital where I practice ‘will somebody submit their resume’?

This complaint about pay was made by Lina:

The tariff they pay us as occupational therapists is minimal.

While Laura commented:

It is probably why a lot of people flee, the responsibility they have and the little pay for all the time they dedicate.

Valentina stated that she had been tempted to abandon her work with stroke survivors in Colombia:

This is pure love in Colombia. I love clinical work, and I can’t imagine doing anything else, but I was in the United States in December of last year and an article came out where it said (occupational therapy) was the sixth highest paid profession at the moment and I said ‘what can I do to come here?’.

The demotivating effect of poor remuneration was also felt by Laura:

I prefer to stay in my house with my family than to go on a home visit for forty or fifty thousand pesos.

### Action/way forward

Daniela, Candelaria and Consuelo all stressed the importance of research in understanding and changing the health system. Daniela called for:

Field research, not in the classroom, in the field. . .based in the research of the problems that we have as professionals.

Candelaria commented that such field research needed to be done in ‘zones where there has been conflict’:

I think that there are zones where we should strengthen a lot, beginning with a lot of things, from the academy sending students and professors doing quite strong field work, research in these zones.

Carmen described a university project in a locality of Bogotá and pointed out that:

There are people. . .for example, the people with stroke that didn’t return to work, they were recluses in the house. . .sometime families with six or seven people in very precarious health conditions and in this moment, I felt really bad, ‘why’, I said, ‘is this Bogotá?’ We don’t even have to go to rural areas, go to a poor municipality to see this, it is here. . .I think we have to adjust more to the reality and research because there is a lot to research here.

In addition, the issue of political participation was raised. Candelaria commented that:

Another challenge, apart from the academic one, is to develop at a higher level, health policy.

Rosa stated this need for participation at a more general level:

I think that we have to move not only in the hospital, not only on the healthcare side, but also as social leaders that we are, we have to go further.

Carmen recalled Colombian occupational therapists’ history in this regard:

Occupational Therapy has a very long history of hard work, of struggle in all the fields, above all in the legal field, in public health and so on. They achieved a lot of victories. I was from the Colombian Association of Occupational Therapy, I was the first representative in the National University, I am speaking here of 1973. It is never-ending work.

A more confrontational stance was supported by Daniela:

. . .I think that we need to move, to motivate the students and everyone who knows the laws and has this posture as a belligerent.

Candelaria stated that:

On the other hand, and this is an area of great academic competence, it is to create this necessity in the students to educate themselves politically and participate in all the discussions, because I think that this a very strong weakness. Very few students and professionals dare, for example, to enter into the rooms of national politics.

The focus group two participants emphasised the need to develop a professional association. Alejandra, for example, stated:

A challenge that is also important is that we are not united as a professional association, and if we do not unite everyone is doing their own things separately, but if we do unite, we could do much bigger things with the stroke population.

This point was echoed by Lina:

. . .we are losing as well, but it’s because we are very disunited as a professional association. We are failing to unite more as a professional association.

Alejandra went on to say:

I think that the day we say, we can continue strengthening ourselves as a professional association, we can obtain many more things, not only with stroke patients, but in general, in all of the areas we intervene in.

## Discussion

The issue of premature discharge was highlighted by several participants. As shown above, there is a lack of data about the Colombian stroke rehabilitation (Ouriques Martins et al., 2019). In the absence of such data, our research makes an important contribution in revealing premature discharge as an issue impeding the work of Colombian stroke occupational therapist, and one that warrants further research.

Participants also discussed the role of the Colombian health insurance system. The EPS provides health plans, however the packages vary and there is no guarantee that occupational therapy will be offered (García Ruiz, 1998). This supports the participants’ views that the EPSs frequently have a negative impact on the services that they are able to offer to stroke survivors.

The issue of lack of awareness of occupational therapy by other health professionals was mentioned by participants. While there are no specific data available for Colombia, researchers have stressed that this is a general problem for the profession, and may be worse in low-to-middle-income countries (Darawsheh, 2018). Darawsheh (2018: 3), for example, found that, in a survey of 829 Jordanian health professionals and ‘lay people’, 45.5% of respondents had no knowledge of occupational therapy. Similarly, it has been found that, in a survey of Nigerian health professions undergraduates, less than 40% had a ‘good knowledge’ of occupational therapy, and 49.5% of medical undergraduate’s knowledge was poor (Olaoye et al., 2016: 4). Again, our research has contributed in revealing that this may be a problem for occupational therapists engaged in stroke rehabilitation in Colombia.

It is important to note that some participants reported positive experiences of hospital-based stroke services. The ‘centre of excellence’ described by two occupational therapists, for example, was distinguished by early assessment, strong teamwork and respect for their professional judgement. This was in contrast to other services, where participants reported premature discharge of stroke survivors and clinical leaders’ and managers’ ignorance about their role.

A number of participants complained that occupational therapists were not being attracted to clinical work, often as a result of poor remuneration. Certainly, there seems to be a problem of precarious employment and low pay among Colombian occupational therapists. For example, Moreno-Chaparro et al. (2022) surveyed 382 occupational therapists in the country and found that 11% had lost their job due to the COVID-19 pandemic, with 38% of this group reporting that their institution had closed and 28.5% that their contract had not been renewed as result of lockdown changes. Reyes (2022) also highlights the levels of job insecurity experienced by occupational therapists in the country. These factors perhaps explain the participants’ comments that occupational therapists may be attracted to other areas of work. We are not aware of data on the career trajectory of registered occupational therapists, and research in this area should be a priority for future work.

It is interesting that one participant reported their preference for working in the United States (US). This tendency has been confirmed by the World Health Organization (WHO), which showed that recruitment of health professionals from lower resource environments by more highly resourced countries was a key problem for the health systems of the lower resourced regions (WHO, 2016). The WHO (2016) has called for improvements in salaries, stable payment and enhanced promotion opportunities for health personnel in low- and middle-income countries as a response to this pressure. It is interesting to note, therefore, that the Colombian Policy for Integral Health Attention stresses the importance of transforming incentives for human talent from a productivity focus to improving health results and enhancing personal and professional development (Ministerio de Salud y Protección Social, 2016).

The theme of developing a professional association was brought up by some participants. El Colegio Colombiano de Terapia Ocupacional was founded in 1972 (Reyes, 2022). However, several newer groups have sought to represent different issues and interest groups within the profession. These include La Asociación de Facultades de Terapia Ocupacional, representing deans and other academic leaders, and El Sindicato Nacional de Profesionales de Fisioterapia, Fonoaudiología y Terapia Ocupacional, which has a trade union focus and seeks to improve the working conditions of therapists in response to economic crisis and deterioration in health services (García Ruíz, 2016). However, no evidence has been identified about the existence of a specialist association for occupational therapists working in stroke rehabilitation. In addition, the participation of occupational therapists in policy-making, which participants mentioned in relation to the development of a professional association, is regarded as an ongoing challenge (Guzmán-Suárez, 2019).

One participant looked back to the period from 1970 to 2000 as a high water mark for the advancement of the profession. It is certainly the case that the profession achieved several victories around this time. The first Colombian occupational therapy training course, for example, started at The National University of Bogotá in 1966, in recognition of the need to establish comprehensive rehabilitation services following the period of political conflict known as ‘La Violencia’ (Rodríguez Mendoza et al., 2017). In addition, Colombian occupational therapy moved from being a technical qualification to a professional degree in 1976; crucially, this occurred after 3 years of campaigning by students and academics, suggesting that one participant’s call for occupational therapists to adopt a more ‘belligerent’ stance may be justified (Rodríguez Mendoza et al., 2017). It is also, perhaps, important to point out here that progress has continued beyond this period, with occupational therapy being defined in law once again in 2005 (Guzmán-Suárez, 2019).

The need for research in conflict zones was also highlighted. The impact of the conflict on Colombian occupational therapists is underlined by the findings from a survey of 34 occupational therapists in regional areas of the country, which showed that the key barriers were lack of economic resources, followed by inaccessibility of populations and insecurity due to the presence of armed actors (Gómez-Galindo et al., 2017). This also confirms the need, foregrounded by some of our participants, for research in rural areas, an approach encapsulated in the Colombian concept of ‘territorial occupational therapy’, which involves moving the profession beyond the urban setting (Reyes, 2022). The Decennial Plan of Public Health 2022–2031 emphasises the importance of pursuing a human rights and equity perspective, a differential rights approach, recognising diversity and intersectionality, a gender perspective and ethnicity. Also, it proposes four pillars for public health: social protection, environmental health, culture for life and health and health integrality (Ministerio de Salud y Protección Social, 2022). Despite this, there is a paucity of research on the role of occupational therapists in conflict and post-conflict environments (Peñas Felizzola et al., 2015), and the comments of the participants are therefore apt.

### Critical realism

The themes identified can also be linked to critical realism concepts. The interrelated issues of the dominance of the EPSs, premature discharge and poor remuneration are an example of the way economic and political structural factors shape individual experience (Leung et al., 2022). These factors were ‘empirically manifested’ to the participants in the form of damaging delays in treatment or even the loss of stroke survivors to the service and, in one case, a wish to seek work in the US (Danermark, 2019: 374). The lack of awareness of the role occupational therapy by other professionals in the health network instantiates the way in which social and psychological relationships shape empirical experience (Ellison and Langhout, 2022). In addition, the calls for further research and the aspiration to develop a professional association are expressions of agency that fit with the emancipatory orientation of critical realism (Looker et al., 2021).

### Strengths and limitations

This research is the first to provide data on the experience of Colombian occupational therapists working in stroke rehabilitation. The contribution is timely, as initiatives such as the ‘Declaration of Gramado’ have given impetus to improving outcomes for stroke survivors in Latin America (Saposnik and Hachinski, 2019: 622).

Limitations of the study include the facilitation of the focus groups and identification of themes by a researcher (JO’B) who speaks Spanish as a second language, which may have affected validity. We would argue that this threat was mitigated by collaborative work with KAR, a native speaker of Colombian Spanish, and the use of back-translation during data analysis.

Further limitations included our sampling approach. First, the focus groups were small, which may lead to individuals dominating the discussion, leading to a bias in results (Asquith, 1997). Future work could use larger groups, or alternatively use individual interviews to collect data. Second, effort should be focused on recruiting participants whose main clinical experience in stroke is, or was, outside of Bogotá, particularly in rural regions or areas of conflict and post-conflict. These measures should produce a sample that is more representative of the experience of Colombian stroke occupational therapists as a whole.

### Future areas of work

Future research should focus on the experiences of occupational therapists working in stroke in regional areas of Colombia, especially those places affected by conflict. Also, the experience of occupational therapists in trying to establish a professional interest group for clinicians and researchers working in stroke rehabilitation should be explored. It is also important to research the alternatives to clinical work that are attracting Colombian occupational therapists away from work in stroke rehabilitation.

## Conclusion

Stroke is a major cause of disability in Colombia. However, there is variability in stroke occupational therapy provision, with people from lower resourced backgrounds having less access. In addition, there is a lack of data about stroke services.

We used thematic analysis within a critical realist theoretical framework to explore the experiences of Colombian occupational therapists working in stroke rehabilitation. Key findings included that, despite a more integrated service in previous decades, premature discharge of stroke survivors from hospital and gaps in occupational therapy provision were a frequent experience. One factor was the role of the health insurance system, which dictates how often a patient is seen, or whether they receive occupational therapy. Two participants reported a better experience and described working in a ‘centre of excellence’ in the capital city. Participants also reported a lack of understanding of the occupational therapy role and an attitude that the occupational therapist’s function was to work with children, or in childlike activities. Connected to this were poor remuneration and job insecurity, which some respondents felt discouraged students from applying for posts. A number of ways forward were identified, including research in rural and conflict zones, building a professional association and taking a more campaigning stance in national politics.

The themes fit well with critical realism, which locates personal experience in a socioeconomic framework. In addition, critical realism focuses on amelioration of the situation explored in research.

Limitations of the study include facilitation of the focus groups by a researcher who was speaking Spanish as a second language. Also, the small sample size and recruitment of occupational therapists working mainly in the capital city may be potential sources of bias in the results.

Overall, our research has provided valuable information on a hitherto unexplored area. It also points out priorities for future research.

**Key findings**

- Identified barriers included premature discharge from hospital, misunderstanding of occupational therapists’ role, poor remuneration and the health insurance system.
- Identified ways forward included research, professional associations and a greater political voice.

**What the study has added**

The study is the first to explore the experiences of occupational therapists working in stroke in Colombia and a range of barriers to effective practice were identified.

## References

[bibr1-03080226231178396] Agredo W Martinez DX Gutiérrez DF (2013) Development of a software tool for rehabilitation of the upper limb following stroke. In: Pan American Health Care Exchanges (PAHCE), Medellin, Colombia, pp. 1–4.

[bibr2-03080226231178396] Asquith JAL (1997) The effects of group size on the outcome of focus group sessions. Management Research News 20: 1–15.

[bibr3-03080226231178396] Arenas Duque A Lucumí DI (2019) Caracterización del accidente cerebrovascular en Colombia. Serie Documentos de Trabajo: 63.

[bibr4-03080226231178396] Bhaskar R Hartwig M (2016) Enlightened Common Sense: The Philosophy of Critical Realism. Abingdon: Routledge.

[bibr5-03080226231178396] Bayona-Ortiz H Useche JN Yanez N , et al. (2019) Availability of stroke units in Colombia. The Lancet Neurology 18: 988. 10.1016/S1474-4422(19)30332-1 31609207

[bibr6-03080226231178396] Braun V Clarke V (2006) Using thematic analysis in psychology. Qualitative Research in Psychology 3: 77–101.

[bibr7-03080226231178396] Braun V Clarke V (2023) Toward good practice in thematic analysis: Avoiding common problems and be(com)ing a knowing researcher. International Journal of Transgender Health 24: 1–6. 36713144 10.1080/26895269.2022.2129597 PMC9879167

[bibr8-03080226231178396] Calderón-Chagualá JA Chacón-Peralta H Vergara-Torres GP , et al. (2015) Estudio de la calidad de vida en pacientes tres meses después de un ictus. Revista Mexicana de Neurociencia 16: 5–15.

[bibr9-03080226231178396] Camacho PA Gomez-Arbelaez D Otero J , et al. (2020) Self-reported prevalence of chronic non-communicable diseases in relation to socioeconomic and educational factors in Colombia: A community-based study in 11 departments. Global Heart 15: 35. 32489808 10.5334/gh.792 PMC7218792

[bibr10-03080226231178396] Camacho S Maldonado N Bustamante J , et al. (2018) How much for a broken heart? Costs of cardiovascular disease in Colombia using a person-based approach. PLoS One 13: e0208513. 30566516 10.1371/journal.pone.0208513 PMC6300287

[bibr11-03080226231178396] Charmaz K (2011) Grounded theory methods in social justice research. In: Denzin NK Lincoln YS (eds) The SAGE Handbook of Qualitative Research, 4th edn. Thousand Oaks, CA: SAGE Publications, pp. 359–380.

[bibr12-03080226231178396] Colombian Government (2019) Ley 1955. Available at: https://www.funcionpublica.gov.co/eva/gestornormativo/norma.php?i=93970 (accessed 28 February 2023).

[bibr13-03080226231178396] Danermark B (2019) Applied interdisciplinary research: A critical realist perspective. Journal of Critical Realism 18: 368–382.

[bibr14-03080226231178396] Darawsheh WB (2018). Awareness and knowledge about occupational therapy in Jordan. Occupational Therapy International 2018: 2493584. 29950955 10.1155/2018/2493584 PMC5987337

[bibr15-03080226231178396] Ellison ER Langhout RD (2022) Critical realism methodology as a guiding framework for interdisciplinary theory enrichment: Reflections on a study of empowerment. Journal of Community Psychology: 1–18. DOI: 10.1002/jcop.22919. 10.1002/jcop.22919 35881860

[bibr16-03080226231178396] Etikan I Abubakar Musa S Alkassim RS (2016) Comparison of convenience sampling and purposive sampling. American Journal of Theoretical and Applied Statistics 5: 1–4.

[bibr17-03080226231178396] García Ruiz AS (1998) La terapia ocupacional en el marco de la seguridad social en salud. Revista Ocupación Humana 7: 63–74.

[bibr18-03080226231178396] García Ruíz S (2016) La necesidad del trabajo en red. Cincuenta años ocupando contextos, transformando vidas. In: Memorias del XVI Congreso Colombiano de Terapia Ocupacional. Medellín, Colombia, pp. 125–126.

[bibr19-03080226231178396] Gómez-Galindo AM Peñas-Felizzola OL Parra-Esquivel EI (2017). Experiencias de terapia ocupacional para la paz: Aportes desde las regiones colombianas. Revista de Salud Pública 19: 664–670. 30183815 10.15446/rsap.V19n5.6248

[bibr20-03080226231178396] Gómez-Restrepo C Tamayo-Martínez N Buitrago G , et al. (2016) Violencia por conflicto armado y prevalencias de trastornos del afecto, ansiedad y problemas mentales en la población adulta colombiana. Revista Colombiana de Psiquiatría 45: 147–153. 27993250 10.1016/j.rcp.2016.11.001

[bibr21-03080226231178396] Guerrero Agámez D Pestana Utria G Diaz Arrieta B , et al. (2021) Mortalidad por enfermedades cerebrovasculares en Colombia: 30 años de observación. Acta Neurológica Colombiana 37: 173–188.

[bibr22-03080226231178396] Guerrero R Gallego AI Becerril-Montekio V , et al. (2011) Sistema de salud de Colombia. Salud Pública de México 53: s144–s155. 21877080

[bibr23-03080226231178396] Guzmán-Suárez OB (2019) Participación de la terapia ocupacional en políticas públicas de salud laboral: un desafío profesional. Revista de la Facultad de Medicina 67: 703–708.

[bibr24-03080226231178396] Henao-Lema CP Arcos-Rodríguez AV (2020) Discapacidad y determinantes sociales de la salud en personas con enfermedad cerebrovascular, San Juan de Pasto (Colombia). Revista Facultad Nacional de Salud Pública 38: e336697.

[bibr25-03080226231178396] Jawad M Vamos EP Najim M , et al. (2019) Impact of armed conflict on cardiovascular disease risk: A systematic review. Heart 105: 1388–1394. 31138670 10.1136/heartjnl-2018-314459

[bibr26-03080226231178396] Legg L Drummond A Leonardi-Bee J , et al. (2007) Occupational therapy for patients with problems in personal activities of daily living after stroke: Systematic review of randomised trials. British Medical Journal 335: 2–8. 17901469 10.1136/bmj.39343.466863.55 PMC2048861

[bibr27-03080226231178396] Leung D Lee C Wang AH , et al. (2022) Immigrants’ and refugees’ experiences of access to health and social services during the COVID-19 pandemic in Toronto, Canada. Journal of Health Services Research and Policy 28: 34–41. 35971256 10.1177/13558196221109148 PMC9382571

[bibr28-03080226231178396] Marín JJ (2006) Reubicación y restablecimiento en la ciudad. Estudio de caso con población en situación de desplazamiento. Universitas Humanística 62: 143–168.

[bibr29-03080226231178396] Ministerio de Salud y Protección Social (2016) Política de Atención Integral en Salud. Available at: https://www.minsalud.gov.co/sites/rid/Lists/BibliotecaDigital/RIDE/DE/modelo-pais-2016.pdf (accessed 28 February 2023).

[bibr30-03080226231178396] Ministerio de Salud y Protección Social (2022) Resolución número 1035 de 14 de junio de 2022. Available at: https://www.minsalud.gov.co/Normatividad_Nuevo/Resoluci%C3%B3n%20No.%201035%20de%202022.pdf (accessed 28 February 2023).

[bibr31-03080226231178396] Moreno-Chaparro J Calderón-Calvo A Cubillos-Mesa C , et al. (2022) Impact of the COVID-19 pandemic on the professional clinical practice of occupational therapy. Cadernos Brasileiros de Terapia Ocupacional 30: 1–14.

[bibr32-03080226231178396] Olaoye OA Emechete AAI Onigbinde AT , et al. (2016) Awareness and knowledge of occupational therapy among Nigerian medical and health sciences undergraduates. Hong Kong Journal of Occupational Therapy 27: 1–6. 30186055 10.1016/j.hkjot.2016.02.001 PMC6091994

[bibr33-03080226231178396] Ouriques Martins SC Sacks C Hacke W , et al. (2019) Priorities to reduce the burden of stroke in Latin American countries. The Lancet Neurology 18: 674–683. 31029579 10.1016/S1474-4422(19)30068-7

[bibr34-03080226231178396] Peñas Felizzola OL Gómez Galindo AM Parra Esquivel EI (2015). Participación de terapia ocupacional en contextos de conflicto armado y postconflicto. Revista de Salud Pública 17: 612–625. 28453080 10.15446/rsap.v17n4.53047

[bibr35-03080226231178396] Reyes MS (2022) Los cincuenta años del Colegio Colombiano de Terapia Ocupacional en un país en transición. Revista Ocupación Humana 22: 184–187.

[bibr36-03080226231178396] Rodríguez Mendoza L Camargo Carrero N Escobar Jiménez X (2017) Terapia ocupacional: una perspectiva histórica desde la Universidad Nacional de Colombia (1966–1989). Revista Ocupación Humana 16: 26–45.

[bibr37-03080226231178396] Royal College of Physicians (2016) National Clinical Guideline for Stroke. Available at: https://www.rcplondon.ac.uk/guidelines-policy/stroke-guidelines (accessed 1 March 2023).

[bibr38-03080226231178396] Saposnik G Hachinski V (2019) The pathway towards an effective reduction of stroke burden worldwide: Teamwork. The Lancet Neurology 18: 622–623. 31202465 10.1016/S1474-4422(19)30198-X

[bibr39-03080226231178396] World Health Organization (2016) Global Strategy on Human Resources for Health: Workforce 2030. Geneva: World Health Organization.

[bibr40-03080226231178396] Yanez N Useche JN Bayona H , et al. (2020) Analyses of mortality and prevalence of cerebrovascular disease in Colombia, South America (2014–2016): A cross-sectional and ecological study. Journal of Stroke and Cerebrovascular Diseases 29: 104699. 32127257 10.1016/j.jstrokecerebrovasdis.2020.104699

